# Athletic Trainers’ Perceptions of Responsibilities and Use of Psychosocial Interventions for Patients Following an ACL Injury

**DOI:** 10.3390/ijerph20186762

**Published:** 2023-09-15

**Authors:** Joshua K. Matthews, Kayleigh A. De Koker, Zachary K. Winkelmann

**Affiliations:** 1 Darla Moore School of Business, University of South Carolina, Columbia, SC 29208, USA; jkm10@email.sc.edu; 2 Department of Exercise Science, University of South Carolina, Columbia, SC 29208, USA; kdekoker@email.sc.edu

**Keywords:** mental health, knee, sports medicine

## Abstract

Following an anterior cruciate ligament (ACL) injury, mental health challenges are often concomitant with the injury and rehabilitation process. Athletic trainers are essential components within the healthcare team who should be trained in recognizing, referring, and managing mental health issues. However, more research is needed on the athletic trainer's responsibility regarding psychosocial interventions and their role within ACL patients. Our descriptive study included 153 collegiate athletic trainers who reported on previous training and responsibilities related to mental health. Of these participants, 98% reported caring for an ACL patient within the last year. The participants were further asked to explore what behavioral responses were observed within ACL injury patients, the specific psychosocial interventions deployed, the frequency of integration, and whether a referral to another provider was utilized. We identified that athletic trainers share a strong understanding of their perceived roles, with 99.3% of participants stating the obligatory feeling to support ACL patients experiencing mental health challenges and implementing personalized rehabilitation (74%) and attainable goals (70%) while also keeping the athlete involved in the team (72%). Our data suggest that athletic trainers recognize their role and continue to integrate psychosocial strategies throughout the ACL injury process.

## 1. Introduction

The anterior cruciate ligament (ACL) is one of four ligaments within the knee that stabilizes and establishes proper knee functionality. However, the ACL can be torn or ruptured in a variety of ways, including sharp turns while running or direct physical contact. This lower extremity health condition occurs between 100,000 and 200,000 times yearly, and the peak age for such an injury is 16 for women and 17 for men [1,2]. A tear of the ACL is most prevalent within the athletic population, with 53% of ACL injuries being sustained during football [3]. An ACL injury is characterized by a lack of stability within the knee, significant edema following the injury, as well as an audible pop or crack which the athlete often reports to the athletic trainer. To maximize knee stabilization and functionality, the most common standard of care for an ACL injury, especially for athletes who want to return to athletic participation, is to undergo the surgical procedure of an ACL reconstruction (ACLR) [4]. In ACLR, the most common method of repair is to utilize grafts that are biomechanically similar to the native ligament that was injured. The bone–patellar tendon–bone graft has historically been considered the “gold standard” for ACLR due to its excellent clinical results and high patient satisfaction [4]. However, the procedure is invasive and creates extensive physical trauma for the patient. Conservative management is useful within the sedentary population; however, this method of treatment is associated with substantial drawbacks when an athlete is not able to resume their high-level athletic ability and includes an increased risk of meniscal and articular cartilage injuries followed by chronic knee instability [5].

Recovery from an ACL injury requires significant physical and mental rehabilitation. Following an ACL injury or ACLR, one of the most commonly cited effects was fear of reinjury [6] or increased fear-avoidance beliefs [7]. Other negative mental health symptoms such as anger and low self-confidence are also commonly self-reported during the rehabilitation process [8]. The most common theory for this phenomenon is that the abrupt change in lifestyle from active to sedentary is a mental shock [9]. Moreover, continued mental challenges can lead to mental illnesses such as anxiety, post-traumatic stress disorder, and depression [10]. Major depressive disorder is characterized by a wide variety of symptoms, some of which may not affect every individual. Depression affects approximately 6.7% of today’s adult population, with a higher prevalence rate for young adults [11]. A recent study on collegiate athletes identified that 22.3% of student-athletes are at risk for depression [12]. The symptoms of depression include feeling sad or anxious, loss of interest in activities, appetite, and weight changes, changes in sleep patterns, decreased energy levels, feelings of worthlessness, trouble concentrating or making decisions, and suicidal thoughts or even actions [13]. Previous research analyzing the depression symptomology for patients who have experienced an anterior cruciate ligament injury showed that the incidence of depression in patients undergoing an ACL reconstruction may be as high as 42% [9]. Similarly, another research study that compared the differences in emotional responses among athletes who sustained a concussion compared to an ACL tear found that athletes with ACL injuries have much more severe levels of depression and a longer duration of symptoms than those with concussions [11]. These symptoms, when left untreated, can last from weeks to years. Each patient who suffers an ACL injury will present differently in their psychosocial response; however, these negative behavioral symptoms have been found to last for up to 2 years, peaking in the first six weeks after surgery and then decreasing during the rehabilitation process.

Athletic trainers work closely with their patients from the time of injury through the rehabilitation process to return them to their activity. An athletic trainer, with their vast educational training and availability, is one of the major supports that an injured athlete relies on during the injury and return-to-activity process. The literature also states that injured athletes received social support from athletic trainers during their recovery process in more than 80% of injury events [14]. Those athletes who reported higher levels of satisfaction with the social support from their athletic trainers during recovery were significantly less likely to experience symptoms of depression and anxiety [15]. Many athletic trainers recognize the mental health challenges an injured athlete may experience and are willing to provide rehabilitation interventions not only for the physical injury but for the psychological repercussions as well. Athletic trainers have shown the ability to mitigate negative mental health consequences, with around 53% of athletes who received mental health support from athletic trainers being highly satisfied with their care. While the mental health support from the athletic trainers did not prevent the initial negative mental health effects, those athletes who were highly satisfied with their care were 87% more likely not to suffer depression symptoms at the time of their return to the sport than those athletes who were not highly satisfied with their support [14].

The scope of athletic training states that an athletic trainer cannot provide a specific behavioral health diagnosis, counseling, or therapy. However, they are equipped to provide and implement interventions to support mental wellness. Athletic trainers are needed to listen, show understanding of the specific injury such as an ACL tear, and provide the necessary psychosocial interventions. With the known risk factors of mental health for student-athletes that predispose this population to a higher rate of depression and other mental health challenges, specifically following an ACL injury, athletic trainers must explore their background, training, and recognition of changes in mental health to provide the best care throughout the injury process. In addition, it is key that we explore athletic trainers’ support mechanisms including the specific interventions implemented, the situations that called for a referral, and the inventions that were deemed unnecessary. However, there is a gap in the literature about what the psychosocial response is in athletes and how athletic trainers provide support. Therefore, this research study aimed to explore the mental health management coordinated and/or delivered by athletic trainers holistically and specifically to patients who have sustained an ACL injury. Due to the descriptive and exploratory nature of the study design, there is no hypothesis. The data will help to inform the sports medicine field regarding the state of training and implementation of psychosocial skills relative to athletic training clinical practice and, more specifically, for patients who have sustained an ACL injury.

## 2. Materials and Methods

To explore the research question, we designed a quantitative, cross-sectional study using an anonymous, web-based survey (Qualtrics Inc., Provo, UT, USA). The study was deemed exempt by the University of South Carolina Institutional Review Board.

### 2.1. Participants

The target population for this study was athletic trainers working in a college/university setting and providing patient care regularly. During the survey, a specific exclusion criterion asking if the participant had managed a patient’s ACL injury or reconstruction in the past year was presented. Only participants with this experience were provided the questions specific to patient implementation. The participants were on average 37 ± 11 years old (range = 23–69 y), were mid-career providers (experience = 14 ± 10 y; range = 1–43 y), and had 12 ± 9 years of experience in a college/university job setting. The majority of participants were women (n = 94, 61.4%) with another 37.9% self-identifying as men (n = 58) and 1 person (0.7%) preferring not to report their gender. Table 1 provides an overview of the governing bodies at the college/university where they provide care.

### 2.2. Instrument

The survey contained an investigator-created instrument that explored multiple aspects of psychosocial skill implementation. The instrument specifically explored the athletic trainers training relative to mental health (three questions), perceptions on the responsibility of the athletic trainer (seven questions), and their background relative to mental health patient care (three questions). After this, we asked participants if they had specifically taken care of a patient for an ACL injury or reconstruction in the past year. If so, they were presented with follow-up questions. These questions asked the participants to indicate what psychosocial responses they have observed in patients following an ACL injury (21 options). Next, we asked them to report how often they deployed certain psychosocial intervention strategies as an athletic trainer (18 options). Finally, they were asked, during the management of patients with an ACL injury and/or reconstruction, which specific mental health interventions and strategies they had either integrated themselves and/or referred to another provider for (30 options). The participant reported their answers for the final section as something they did, something they referred out for, or something they did not do, with an explanation as unaware, unable to do, or unwilling to implement based on the pre-contemplation behavior theory subscales.

The survey questions were adapted from previous literature on mental health and ACL injury responses [16]. Before data collection, the research team solicited feedback from three content experts on mental health and athletic training. The experts were three women who were certified athletic trainers and peer-reviewed publications on mental health topics and/or ACL injury response. The feedback process followed a content validation index process [17]. After three rounds of feedback, we achieved a content validation index of 1.0 for both the relevance and clarity of the questions. The index score exceeded the acceptable content validation score needed in the literature, suggesting the instrument had achieved content validity [17].

### 2.3. Procedures

We recruited 4000 college/university athletic trainers from the National Athletic Trainers’ Association Research Database. To recruit the providers, an invitation to participate was sent via email on 3 November 2022. The invitation included a link to the web-based survey where the individual provided electronic acknowledgment of their informed consent to participate and confirmed their role as an athletic trainer in the job setting currently providing direct patient care. Three recruitment email reminders were sent to the same pool of potential participants on 10 November, 1 December, and 8 December 2022 before the survey was closed after being open for 6 weeks.

In total, 177 individuals accessed the survey (access rate = 4.4%); however, 24 individuals were removed from the survey as they either were not certified and providing patient care (n = 18), did not consent to participate (n = 1), or completed less than 70% of the survey (n = 5). The final sample of participants included in the analysis was 153 athletics trainers, which included 23 incomplete responses (71–95% of the survey completed) and 130 who completed the entirety of the survey. Based on prevalence statistics recommendations for a target finite population of 4000 people with an 8% margin of error (confidence interval), we needed a sample size of 145 people, which our study achieved [15].

### 2.4. Data Analysis

Data were downloaded from the web-based survey. The data were collected anonymously through Qualtrics before being exported to SPSS (IBM, Version 28, Armonk, NY, USA) for all statistical analyses. Data were cleaned to remove all participant responses who completed 70% or less of the survey. Data from participants who completed 71–95% of the survey were individuals who left the survey early rather than skipped questions/items. It is common practice to include partial responses in descriptive survey research as the participant has the right to leave the electronic survey at any time. Next, the responses were analyzed using descriptive statistics (mean, standard deviation, and frequencies) for demographics and all variables of interest.

## 3. Results

### 3.1. Background and Training

Overall, 89.5% (n = 137/153) of athletic trainers stated that they had received training or education on how to identify patients with behavioral health conditions, with 84.3% (n = 129/153) of them determining that the education or training was helpful. Similarly, 92.1% (n= 141/153) of survey subjects had received training or education on how to refer patients with behavioral health concerns. Interestingly, 16.3% of respondents (n = 25/153) stated they had never received training or education on how to give support to a patient with a behavioral health concern, and only 73.2% (n = 112/153) stated they had received helpful education on this process. In total, 66.0% (n = 101/153) had evaluated a patient for a mental health concern at their current job, with 72.5% (n = 11/152) having managed a patient at their current job for a mental health concern and 95.4% (n = 146/153) having referred a patient with a mental health concern at their current job.

### 3.2. Perceived Responsibilities of Athletic Trainers in a Mental Health Situation

The most agreed upon topics were whether or not to support patients experiencing mental health challenges, with 99.3% (n = 152/153) agreeing that it was the responsibility of an athletic trainer, as shown in Table 2. Similarly, 96.1% (n = 147/152) agreed that athletic trainers should facilitate mental health referrals, while 94.1% (n = 144/153) agreed with the responsibility of working collaboratively with other healthcare professionals to monitor these patients’ treatment and progress. A total of 81.7% (n = 125/153) of the athletic trainers surveyed stated they felt it was within their duties to identify patients with mental health conditions, and 81.0% (n = 124/153) felt that they should develop and implement specific policies and procedures for mental health. Several categories were fairly uniformly identified as not lying within the responsibilities of an athletic trainer. These were the duty to provide mental health counseling, with 83.0% (n = 127/153) of survey subjects saying no, and 56.9% (n = 87/153) of survey participants saying no to implementing psychological interventions.

### 3.3. Psychosocial Responses Following ACL Injury

It was noted that during the past year, 98% (n = 150/153) of athletic trainers reported having managed an ACL injury and/or reconstruction. Overall, the athletic trainers who managed a patient’s care for an ACL injury reported that the most common psychosocial response in nearly every patient included anger and frustration (42.7%, n = 64/150) or stress (38.0%, n = 57/150). Interestingly, combining the data observed in “several”, “more than half”, and “nearly every” patient resulted in high occurrences of the fear of reinjury (95.4%, n = 144/149) and nervousness returning to a sport (93.3%, n = 140/150). The responses most commonly reported as not observed in patients following an ACL injury included suicidal thoughts (82.7%, n = 124/150), substance misuse (71.35, n = 107/150), and disordered eating (64.7%, n = 97/150). The athletic trainers reported a lack of motivation (61.3%, n = 92/150), lack of attention or concentration (54.0, n = 81/150), isolation/withdrawal (59.3%, n = 89/150), mood shifts (55.3%, n = 83/150), and pain catastrophizing (51.3, n = 77/150) in over half of the patients within their care during the recovery from an ACL injury. Table 3 provides an overview of the psychosocial responses reported by athletic trainers post-ACL injury.

### 3.4. General Psychosocial Strategies Used in Patient Care

The athletic trainers reported the psychosocial strategies they implemented to address the mental health concerns of patients, as shown in Table 4. The athletic trainers reported that the most common psychosocial strategies used included personalizing rehabilitation (74%, n = 112/150), keeping the patient involved with the team (72%, n = 108/150), setting realistic goals (70%, n = 135/150), addressing fear of reinjury (64%, n = 97/150), facilitating understanding of injury (64%, n = 96/150), and using active listening (63%, n = 95/150). The psychosocial strategies most commonly reported as never used included teaching emotional control strategies (23%, n = 35/150) and teaching concentration strategies (23%, n = 35/150). Over 70% (n = 112/150) of the athletic trainers reported that they personalized rehabilitation and set realistic goals, but only 48% (n = 72/150) always understood the individual’s motivation. Only 24.0% (n = 36/150) of participants reported always using strategies to reduce stress, despite stress being one of the most common psychosocial effects noted in the survey.

### 3.5. Specific Mental Health Interventions and Strategies for Patients with an ACL Injury

Table 5 shows the full data for the psychosocial interventions that athletic trainers used. The most common activities or exercises that athletic trainers themselves implemented included confidence-building activities, with 87.2% (n = 123/141) of responses saying they implemented this activity. The next two most commonly used exercises were changing a patient’s self-talk at 69% (n = 100/145) and stress response and management at 58.2% (n = 85/146). There were also a variety of exercises or activities in which the athletic trainers surveyed most commonly referred out to a specialist. The treatments most referred out for included psychiatric services, supportive therapy/counseling, and cognitive behavioral therapy. Supportive therapy/counseling was the most commonly referred treatment, with 70.9% (n = 97/137) of athletic trainers referring patients needing this treatment to others. Psychiatric services followed with 65.2% (n = 86/132), while cognitive behavioral therapy had a lesser referral amount at 49.6% (n = 66/133). It is important to note, however, that cognitive behavioral therapy was not being implemented by the athletic trainers themselves, with 38.8% (n = 53/133) of athletic trainers saying that they were not aware of this intervention taking place or that they were unable to implement it. There were several treatments that athletic trainers most commonly said they were unaware of as being used. These included impulse control, with 36% (n = 45/125) of those surveyed stating they were unaware of it being used in treatment. Similarly, 37.6% (n = 50/133) of athletic trainers stated that they were unaware of exposure therapy being used, and 48.5% (n = 65/134) of the athletic trainers said the same about creativity and art therapy. In total, 31.4% (n = 42/134) of athletic trainers stated that they were unable to implement creativity and art therapy as a specific intervention. Several other specific interventions were also cited most commonly as unable to be implemented, such as energy therapy at 33.6% (n = 43/128), acupuncture at 38.9% (n = 49/126), and essential oils and aromatherapy at 33.6% (n = 42/125). Essential oils and aromatherapy were specific interventions that many athletic trainers refused to implement, with 23.2% (n = 29/125) of those surveyed reporting that they would not choose such an exercise. The only other specific interventions that were heavily cited as refused to implement were herbal supplements at 27.4% (n = 34/124) and relaxation therapy, with 51.5% (n = 67/130) of athletic trainers saying they would refuse to implement it.

## 4. Discussion

This research supports the growing evidence that the prevalence of anterior cruciate ligament tears within sports is high and often treated with reconstruction surgery, as this is the gold standard treatment [9]. Anterior cruciate ligament tears are an extremely common injury with an occurrence rate in athletes growing annually [18]. Due to these injuries, up to 40% of patients experience major depressive disorder symptomology over the course of their rehabilitation [9]. Symptoms of depression can include sadness, anxiety, hopelessness, and lack of sleep. These symptoms all hinder a patient’s ability to recover mentally and physically from their injury. Similarly, a limited number of athletes who have sustained an ACL injury that return to sport at a competitive level after an ACL tear cite mental limitations such as fear of reinjury [6]. Our data support this conclusion as fear of reinjury was one of the highest psychosocial responses that athletic trainers identified in their patients. Similarly, an ACL injury and the resulting treatment have been known to be a factor in the mental health of patients, with up to half presenting with clinically relevant depression [19]. Regarding this, the literature emphasizes the role of the athletic trainer as an important source of emotional support since they are equipped with the necessary skills and knowledge to rehabilitate the athlete through their physical disability but also support their mental challenges [14]. Almost all of the athletic trainers surveyed deployed strategies to reduce depressive symptoms, providing evidence for the common recognition of the role traumatic injuries like ACL tears play in a patient’s mental health. Our data support the notion that athletic trainers can recognize, refer, and provide additional support within their scope of training to combat negative psychological responses following an injury [14].

### 4.1. Background and Training

There are multiple members of the Athletic Training Strategic Alliance who provided insight into some of the discrepancies between training and the current standard of care relative to mental health. The Commission on Accreditation of Athletic Training Education (CAATE) focuses on developing and accrediting athletic training education, while the National Athletic Trainers’ Association focuses on the growth of the profession and athletic trainers as unique healthcare providers [20]. Because the idea of athletic trainers bearing responsibility in the rehabilitation of the psychosocial symptoms of patients is considered newer, this may explain the 16.3% of athletic trainers who stated that they have not received training in how to give support to a patient with a behavioral health concern. However, for years, the National Athletic Trainers’ Association has had a requirement of 12 areas in which athletic trainers must be clinically proficient, which include the domain of psychological intervention and referral. This psychological intervention and referral competency focuses on communication, motivation and adherence strategies, social support and basic counseling skills, mental skills training, and potential referral situations [21]. It is important to note that the role of athletic trainers as defined by this competency concerning psychological health is focused on intervention and referral, not prevention. As such, athletic trainers should not be expected or trained to prevent psychosocial responses to injury, especially without current education.

Currently, the CAATE standard which is used to educate and train athletic training students both didactically and clinically states that the student should be able to “identify, refer, and give support to patients with behavioral health conditions. Work with other health care professionals to monitor these patients’ treatment, compliance, progress, and readiness to participate” [20]. The focus should instead be on recovery and, when needed, referring patients to specialists in the psychological field. This supports our data which show that a strong majority of athletic trainers (96.1%) reported having been trained in how to refer patients with behavioral health issues. However, a small minority of athletic trainers (16.3%) reported they had never received training or education on how to give support to a patient with behavioral health concerns. This is supported by research conducted on how comfortable athletic trainers feel communicating with patients and other physicians, showing that communication with patients was rated a 4.3 out of 10, while communication with other physicians was 6.7 out of 10 [21]. This suggests that one of the key limiting factors in the psychosocial care an athletic trainer can provide is simply their ability to effectively communicate with their patients. In research from 2105, athletic trainers reported their perceived responsibilities of psychosocial competencies to mostly include facilitating a referral (97.3%). Interestingly, our data suggest that more athletic trainers are aware of their scope of practice and training, with a move from 36% of athletic trainers in 2015 stating it was their responsibility to provide counseling to less than 10% in our study indicating it was their responsibility [16]. This is a positive move for athletic trainers recognizing their role in mental healthcare. However, we identified that fewer athletic trainers feel they can implement psychological interventions, a change from 43.0% in 2015 to 23.5% in our study [6].

The scope of training for an athletic trainer includes providing support to patients dealing with mental health challenges; although this is an ambiguous term, support can include creating awareness within the team about mental health specifically within the context of injuries to creating personalized rehabilitations. In addition, psychological interventions must be individualized, focused on the patient’s well-being, and curated to not hinder their ability to progress through the rehabilitation process but rather encourage patients while also treating their psychosocial responses. The educational background of athletic trainers does not include the ability to diagnose or treat specific mental illnesses; therefore, interprofessional collaboration within a healthcare team is imperative to ensure that once the signs of depression or another mental health issue surface, there is an efficient method of supporting and then referring the patient to the necessary care. According to previous research on the roles of athletic trainers and mental health, it was determined that “if student-athletes demonstrate or voice an imminent threat to themselves, others, or property, report feeling out of control or unable to make sound decisions; or are incoherent or confused or express delusional thoughts, emergent mental health referral is recommended [22]”. This suggests that athletic trainers should look to provide mental support to patients when symptoms do not reach such severity, stating that education on possible mental conditions due to injury, as well as early recognition of signs of mental health issues, is critical in the treatment of these issues. We believe athletic trainers need to continue developing their knowledge of psychological ,rehabilitation while also developing their understanding of the difference between psychosocial interventions that can provide support from psychiatric services and counseling. One mechanism to do so is advanced practice training in mental and behavioral health such as specialty certificates and residency programs.

### 4.2. Perceived Responsibilities of Athletic Trainers in a Mental Health Situation

The perceived responsibility of the athletic trainers varied, but our data indicated that each potential responsibility had a strong majority in either the yes or no categories, with very few athletic trainers needing clarification about their perceived responsibilities. The most commonly perceived responsibilities of the athletic trainers were to identify patients with mental health conditions, facilitate mental health referrals, and support patients experiencing mental health challenges. These data are consistent with previous research suggesting that 84.3% of athletes receive social support from their athletic trainers [14]. Athletic trainers felt that they were not responsible for certain responsibilities, including implementing psychological interventions and providing mental health counseling. Interestingly, research on the confidence level of athletic trainers to implement these responsibilities determined that athletic trainers were only fairly confident in their ability to recognize or refer a patient for their mental health needs. However, the participants were very confident in their ability to refer mental health emergencies [23]. This suggests that while athletic trainers are generally aware of their roles and responsibilities in mental health situations, there is a disconnect between the ability to identify the mental health issue and the perceived responsibility. Importantly, athletic trainers are also much more comfortable reporting and referring patients than they are in facilitating psychosocial recovery themselves. However, while the ability to refer patients when needed is extremely valuable, athletic trainers should be comfortable in dealing with psychosocial symptoms that do not require referral themselves. Combining the data from Table 2 and Table 5, 56.9% of athletic trainers did not feel it was their responsibility to implement psychosocial interventions. The data on the interventions used either identify a lack of knowledge of interventions for mental health or a perceived-to-actual practice gap. For example, of the participants using psychiatric services to help patients with ACL injuries, the majority referred out to another provider (65.2%); however, there are techniques athletic trainers are using in the scope of training such as progressive muscle relaxation, which over 50% (n = 67) of athletic trainers reported using. Previous literature identified that musculoskeletal physiotherapists did not feel properly trained to implement interventions, despite knowing their potential benefits to patients [24].

### 4.3. Psychosocial Responses Following ACL Injury

The psychosocial data suggest that there are a variety of responses with differing prevalence experienced by ACL patients over the course of their rehabilitation. The ability to accept one’s injury, specifically in the two weeks following reconstruction surgery, has positively impacted mental health outcomes, indicating fewer symptoms of depression and less use of alcohol and other negative coping strategies [25]. The athletic trainers in our study reported anger, frustration, stress, anxiety, nervousness about returning to the sport, and fear of reinjury among the most commonly reported psychosocial responses following an ACL injury. These psychosocial responses recognized by athletic trainers are similar to previous research completed on the psychosocial symptoms suffered by patients of orthopedic trauma. There is an evident need to help patients accept their injuries through screening, goal setting, and patient education. The Hamilton score, or HAMA, is an extremely common self-reporting scale used to diagnose anxiety levels [26]. A study utilizing this scale in orthopedic patients found that anxiety and depression were among the most commonly reported mental symptoms post-injury, with a degree that rose based on the level of trauma received [27]. Perhaps due to the prevalence of data showing the value of social support on the mental recovery of injured patients, over 90% of athletic trainers reported that none or only several of their patients had a lack of social support. Another tool that is valuable in determining psychosocial symptoms is the Athlete Fear Avoidance Questionnaire. This patient-reported outcome is used to measure sports injury-related fear in athletes and was developed to determine their levels of fear avoidance [28]. This scale found a high correlation between fear avoidance, depression, and anxiety, which supports the findings of this study that these psychosocial responses are among the most commonly reported by athletes on their path to rehabilitation. Finally, the ACL-RSI, or ACL-Return to Sport Index, is commonly used in athlete rehabilitation from an ACL injury to determine the return to sport after injury and the psychosocial effects. It found that while 91% of athletes expect to return to their previous level of sport at least one year after injury, only 41% ended up doing so [29]. The study utilizing the ACL-RSI also found that the two largest increases in scores were between pre-operation and 4 months, and 6 months and one year. This suggests that the largest increases in a patient’s mental recovery and ability to return to sport occur during the time when these patients are most likely to have significant contact with athletic trainers who are working to help facilitate their recovery, emphasizing the importance of psychosocial support.

### 4.4. Psychosocial Strategies and Mental Health Interventions Following ACL Injury

Specific health interventions and strategies reflect the most commonly cited mental health issues occurring among ACL patients; athletic identity, performance ability, a rigorous schedule, and time commitments all contribute to the added stress of being an athlete, especially at the collegiate level, so that adding an injury into the mix with a prolonged recovery that pulls an athlete away from their sport and team contributes further to their psychological responses [11]. Athletic trainers reported often engaging in psychosocial strategies such as confidence-building exercises, psycho-education, and changing patients’ self-talk on a majority scale. These strategies aligned with the most commonly reported symptoms of patients such as anxiety and fear and are especially valuable when compared to research showing the value of a positive outlook. A positive outlook was cited as one of the most important aspects of patients who cope well with their injuries, while a poor attitude was one of the most significant in patients who did not cope well with their injuries [30]. This emphasizes the value of athletic trainers providing social support for patients with ACL injuries at the collegiate level, as only 55% of athletes return to their previous level of sport, and only 81% return to sport on any level [31]. This gap of 26% could potentially be narrowed by athletic trainers providing social support. To help contribute to a positive environment for the athlete, confidence building is a strategy that could be implemented as it would address the commonly reported psychosocial symptoms of anxiety and fear of reinjury while also helping the patient build a positive outlook. Specific strategies that could bemost athletic trainers and could positively affectf-thought, a strategy that only 52% of athletic trainers reported that they used all the time. Similarly, enhancing self-confidence was not used all the time by a majority of athletic trainers and could have a positive effect on patients’ rehabilitation and return to sport. Goal setting has been shown to increase patients’ self-confidence as well, with patients reporting that goal setting was important to them and that they felt it improved their rehabilitation [32]. Another important piece of social support is the personalization of rehabilitation, with patients reporting that when they felt their athletic trainers cared about them on a social level, there was a positive effect on their rehabilitation [33]. However, in our study, 70% of athletic trainers reported that they implemented personalized rehabilitation yet only 48% reported always understanding the patient’s individual motivation. This suggests that athletic trainers can identify the positive psychosocial effects of patient-centered care, but the implementation may be flawed. Relaxation strategies, which were not commonly reported as being implemented by athletic trainers, are effective at reducing stress in patients when implemented. Specifically, relaxation strategies were shown to lower cortisol levels in athletes who were rehabilitating from an injury, implying that they should likely be more commonly implemented by athletic trainers in patients dealing with a stress response [34].

Interestingly, the data in Table 5 identified a potential self-report issue by athletic trainers. Athletic trainers reported the use of “changing patient’s self-talk” (100, 69%), “psycho-education” (83, 61.5%), and “coping self-statements” (53, 41.1%) as an intervention they personally did with their patients. These are all considered facets of therapy, specifically cognitive behavioral therapy, which is outside the scope of the training and abilities of an athletic trainer. The athletic trainers in this study, however, did self-report referring out for cognitive behavioral therapy (9, 6.8%) which is a promising finding suggesting that cognitive therapy itself requires more training but that the additional information could provide insight that the athletic trainers may be practicing common features of counseling and therapy, thus blurring the lines relative to the scope of practice. Previous research has suggested a lack of concordance with the psychosocial techniques used during patient encounters when assessed using an end-of-session checklist from the patient and provider perspectives [35]. However, this is one study without proper training of the patient’s knowledge of the behavioral checklist items. The process of implementing patient-centered psychosocial strategies will be dependent upon the provider’s respect and values of the patient’s preferences. For example, the aforementioned study explored the use of goals, variety in exercise, improving social support, and enhancing self-confidence which are all things that the prexamining actual behaviors through patient feedback, as performed in the cited study, andective review of one’s clinical practice while also examining actual behaviors through both patient feedback, as performed in the cited study, as well as through direct observation.

### 4.5. Limitations and Future Research

The limitations of this study include the small sample size. The sample size was determined by the number of respondents out of a pool of 4000 over a survey period of six weeks, which limits the generalizability. The data from our study only report the perceived implementation of mental health interventions and psychosocial strategies deployed for patients who suffered an ACL injury, which is a major limitation relative to the actual behaviors during clinical practice. In addition, our tool did not allow for the athletic trainer to report the differences between patients for referral. The rationale for this decision was that the skills should be something they could do or needed to be referred out for, and if they still needed to choose one of those options, we wanted to examine why they were not using it. However, the decision to implement a psychosocial strategy could indeed have differed from patient-to-patient. We suggest that future research address educating athletic trainers on how to identify psychosocial symptoms in patients with and without ACL injuries as well as collect direct observation data through incognito standardized patient encounters to have a more comprehensive grasp on the deployment of these strategies from patient-to-patient in real time [36].

In addition, the next critical step in this line of study involves determining the timing of psychosocial interventions. As the literature supports the development of depressive symptoms following ACL injury, we believe it would be best to implement mental health interventions for all patients while healthy in order to act as a deterrent for potential mental health symptomology. Future research should compare the effects of training all athletes in mental healthcare while healthy compared to those with no training to determine if there is an effect following an injury and their response to that injury. It should also include research on the effectiveness of specific mental interventions implemented by athletic trainers after symptoms are observed in patients with and without mental health training to determine the post-symptomology effectiveness of these treatments.

## 5. Conclusions

Patients who have sustained an ACL injury may encounter a wide variety of psychosocial responses to injury, with the most common including anxiety, fear of reinjury, anger, and frustration. When encountering these responses, based on the data of our sample, collegiate athletic trainers have a strong grasp of their roles in treating and referring when necessary. Collegiate athletic trainers do so by employing a wide range of specific mental health interventions. These interventions are used with various frequency which tends to correlate with the prominence of the psychosocial symptoms reported. However, there is room for growth between the specific interventions used and the symptoms reported, as well as between the perceived roles of athletic trainers and their confidence and ability to fulfill the requirements of these roles. The effectiveness of athletic trainers in treating psychosocial issues in patients with ACL injuries will increase as education and their ability to communicate with patients increases, as it will limit the disconnect between the strategies used and the psychosocial issues that exist.

## Figures and Tables

**Table 1:** Governing bodies.

Division and League	N, %
NCAA Division 1	50, 32.7%
NCAA Division 3	39, 25.5%
NCAA Division 2	25, 16.3%
NAIA	21, 15.0%
NJCAA	19, 5.9%
CCCAA	16, 3.9%
Independent/Other	1, 0.7%

NAIA = National Association of Intercollegiate Athletics; NJCAA = National Junior College Athletic Association; CCCAA = California Community College Athletic Association.

**Table 2:** Perceived responsibilities in a mental health situation.

Role and Responsibility	Yes	No	Unsure
Identify patients with mental health conditions	125, 81.7%	13, 8.5%	15, 9.8%
Facilitate mental health referrals	147, 96.1%	4, 2.6%	1, 0.7%
Implement psychological interventions	36, 23.5%	87, 56.9%	30, 19.6%
Support patients experiencing mental health challenges	152, 99.3%	0, 0%	1, 0.7%
Provide mental health counseling	15, 9.8%	127, 83.0%	11, 7.2%
Work collaboratively with other healthcare professionals to monitor these patients’ treatment and progress	144, 94.1%	4, 2.6%	5, 3.3%
Develop and implement specific policies and procedures for mental health	124, 81.0%	15, 9.8%	14, 9.2%

**Table 3:** Responses following an ACL injury observed by the athletic trainer.

Psychosocial Responses	Missing	None of the Patients	Several of the Patients	More Than Half of the Patients	Nearly Every Patient
Stress	6, 4.0%	6, 4.0%	40, 26.7%	41, 27.3%	57, 38.0%
Anxiety	6, 4.0%	5, 3.3%	50, 33.3%	49, 32.7%	40, 26.7%
Anger and frustration	6, 4.0%	3, 2.0%	30, 20.0%	47, 31.3%	64, 42.7%
Lack of confidence	5, 3.3%	4, 2.7%	40, 26.7%	54, 36.0%	47, 31.3%
Fear of movement	8, 5.3%	25, 16.7%	64, 42.7%	38, 25.3%	15, 10.0%
Lack of motivation	5, 3.3%	21, 14.0%	92, 61.3%	19, 12.7%	13, 8.7%
Lack of attention or concentration	5, 3.3%	30, 20.0%	81, 54.0%	24, 16.0%	10, 6.7%
Pain catastrophizing	5, 3.3%	35, 23.3%	77, 51.3%	25, 16.7%	8, 5.3%
Exercise addiction	5, 3.3%	58, 38.7%	65, 43.3%	19, 12.7%	3, 2.0%
Disordered eating	5, 3.3%	97, 64.7%	44, 29.3%	4, 2.7%	0, 0%
Unwanted thoughts	5, 3.3%	57, 38.0%	63, 42.0%	16, 10.7%	9, 6.0%
Fear of reinjury	5, 3.3%	2, 1.3%	30, 20.0%	60, 40.0%	52, 35.3%
Nervousness returning to sport	9, 6.0%	1, 0.7%	37, 24.7%	67, 44.7%	36, 24.0%
Negative self-labeling	11, 7.3%	49, 32.7%	71, 47.3%	12, 8.0%	7, 4.7%
Depersonalization	12, 8.0%	62, 41.3%	62, 41.3%	10, 6.7%	4, 2.7%
Isolation, withdrawal, and alienation	9, 6.0%	28, 18.7%	89, 59.3%	21, 14.0%	3, 2.0%
Substance use/misuse	9, 6.0%	107, 71.3%	33, 22.0%	1, 0.7%	0, 0%
Suicidal thoughts	10, 6.7%	124, 82.7%	16, 10.7%	0, 0%	0, 0%
Lack of social support	9, 6.0%	69, 46.0%	65, 43.3%	6, 4.0%	1, 0.7%
Not understanding the ACL injury	8, 5.3%	43, 28.7%	65, 43.3%	30, 20.0%	4, 2.7%
Mood shifts	8, 5.3%	16, 10.7%	83, 55.3%	31, 20.7%	12, 8.0%

**Table 4:** Strategies implemented in patient care.

Strategy	Missing	Never Use	Use 25% of the Time	Use 50% of the Time	Use 75% of the Time	Use 100% of the Time
Understanding the individual’s motivation	12, 8.0%	0, 0%	4, 2.7%	21, 14.0%	41, 27.3%	72, 48.0%
Using active listening	13, 8.7%	0, 0%	1, 0.7%	5, 3.3%	36, 24.0%	95, 63.3%
Setting realistic goals	12, 8.0%	0, 0%	0, 0%	3, 2.0%	30, 20.0%	105, 70.0%
Enhancing self-confidence	13, 8.7%	1, 0.7%	3, 2.0%	14, 9.3%	46, 30.7%	73, 48.7%
Encouraging positive self-thoughts	12, 8.0%	0, 0%	2, 1.3%	19, 2.7%	39, 26.0%	78, 52.0%
Reducing stress	12, 8.0%	4, 2.7%	12, 8.0%	38, 25.3%	48, 32.0%	36, 24.0%
Reducing anxiety	13, 8.7%	6, 4.0%	22, 14.7%	38, 25.3%	39, 26.0%	32, 21.3%
Improving social support	12, 8.0%	9, 6.0%	17, 11.3%	32, 21.3%	48, 32.0%	32, 21.3%
Reducing depression	14, 9.3%	17, 11.3%	23, 15.3%	41, 27.3%	38, 25.3%	17, 11.3%
Teaching emotional control strategies	12, 8.0%	35, 23.3%	36, 24.0%	32, 21.3%	23, 15.2%	12, 8.0%
Teaching concentration strategies	12, 8.0%	35, 23.3%	36, 24.0%	31, 20.7%	27, 18.0%	9, 6.0%
Teaching the use of mental imagery	12, 8.0%	22, 14.7%	35, 23.3%	35, 23.3%	25, 16.7%	21, 14.0%
Teaching muscular relaxation strategies	13, 8.7%	21, 14.0%	29, 19.3%	35, 23.3%	31, 20.7%	21, 14.0%
Keeping the patient involved with the team	12, 8.0%	1, 0.7%	2, 1.3%	5, 3.3%	22, 14.7%	108, 72.0%
Personalizing rehabilitation	13, 8.7%	0, 0%	0, 0%	3, 2.0%	22, 14.7%	112, 74.7%
Addressing fear of reinjury	13, 8.7%	0, 0%	5, 3.3%	2, 1.3%	33, 22.0%	97, 64.7%
Addressing nutrition	12, 8.0%	6, 4.0%	22, 14.7%	30, 20.0%	41, 27.3%	39, 26.0%
Facilitating understanding of injury	12, 8.0%	0, 0%	2, 1.3%	10, 6.7%	30, 20.0%	96, 64.0%

**Table 5:** Specific mental health interventions implemented in patient care.

Interventions	Yes—I Did with My Patients	Yes—I Referred Out for This	No—Unaware of Intervention	No—Unable to Implement Intervention	No—Unwilling to Implement Intervention
Confidence-building activities	123, 87.2%	14, 10.0%	2, 1.4%	2, 1.4%	0, 0%
Psycho-education (e.g., normalizing ACL injury)	83, 61.5%	13, 9.6%	25, 18.5%	14, 10.4%	0, 0%
Developing focus-cues	77, 57.1%	8, 5.9%	33, 24.4%	17, 12.6%	0, 0%
Stress response and management	85, 58.2%	48, 32.9%	7, 4.8%	5, 3.4%	1, 0.7%
Changing patient’s self-talk	100, 69%	25, 17.2%	13, 9.0%	5, 3.4%	2, 1.4%
Interpersonal skills	83, 61.5%	16, 11.8%	22, 16.3%	12, 8.9%	2, 1.5%
Coping self-statements	53, 41.1%	26, 20.2%	27, 20.9%	20, 15.5%	3, 2.3%
Anxiety grounding	41, 30.2%	40, 29.4%	32, 23.5%	20, 14.7%	3, 2.2%
Mindfulness	63, 46.7%	22, 16.3%	22, 16.3%	25, 18.5%	3, 2.2%
Psychiatric services	3, 2.3%	86, 65.2%	14, 10.6%	26, 19.6%	3, 2.3%
Journaling	60, 45.1%	23, 17.3%	19, 14.3%	28, 21.0%	3, 2.3%
Crisis response	10, 7.8%	65, 50.8%	21, 16.4%	29, 22.7%	3, 2.3%
Supportive therapy/counseling	15, 10.9%	97, 70.9%	8, 5.8%	13, 9.5%	4, 2.9%
Biofeedback	61, 46.2%	23, 17.5%	18, 13.6%	26, 19.7%	4, 3.0%
Progressive muscle relaxation	67, 51.5%	14, 10.8%	27, 20.8%	17, 13.1%	5, 3.8%
Massage therapy	60, 45.1%	43, 32.3%	7, 5.3%	18, 13.5%	5, 3.8%
Cognitive behavioral therapy	9, 6.8%	66, 49.6%	22, 16.5%	31, 23.3%	5, 3.8%
Impulse control	18, 14.4%	23, 18.4%	45, 36.0%	34, 27.2%	5, 4.0%
Exposure therapy	21, 15.8%	21, 15.8%	50, 37.6%	36, 27.0%	5, 3.8%
Deep breathing and breathing control exercises	81, 61.4%	18, 13.6%	9, 6.8%	18, 13.6%	6, 4.6%
Pharmacotherapy	11, 8.4%	54, 41.2%	27, 20.6%	33, 25.2%	6, 4.6%
Meditation	4, 4.4%	36, 40.0%	8, 8.9%	35, 38.9%	7, 7.8%
Guided imagery	46, 33.6%	17, 12.4%	31, 22.6%	36, 26.3%	7, 5.1%
Energy therapy (Qi Gong, Tai Chi, Yoga, etc.)	16, 12.5%	24, 18.8%	34, 26.5%	43, 33.6%	11, 8.6%
Religion and spirituality counseling and/or prayer	25, 19.8%	38, 30.2%	17, 13.5%	34, 27%	12, 9.5%
Creativity and art therapy (e.g., painting, music, acting, and dance)	7, 5.2%	8, 6.0%	65, 48.5%	42, 31.3%	12, 9.0%
Acupuncture	15, 11.9%	32, 25.4%	18, 14.3%	49, 38.9%	12, 9.5%
Essential oils and aromatherapy	11, 8.8%	7, 5.6%	36, 28.8%	42, 33.6%	29, 23.2%
Herbal supplements	6, 4.8%	21, 16.9%	24, 19.4%	39, 31.5%	34, 27.4%
Relaxation training	15, 11.5%	24, 18.5%	17, 13.1%	7, 5.4%	67, 51.5%

## Data Availability

The data presented in this study are available on request from the corresponding author. The data are not publicly available due to IRB protections.
